# Haplotype frequencies at the DRD2 locus in populations of the East European Plain

**DOI:** 10.1186/1471-2156-10-62

**Published:** 2009-09-30

**Authors:** Olga V Flegontova, Andrey V Khrunin, Olga I Lylova, Larisa A Tarskaia, Victor A Spitsyn, Alexey I Mikulich, Svetlana A Limborska

**Affiliations:** 1 Department of Human Molecular Genetics, Institute of Molecular Genetics, Russian Academy of Sciences, Moscow, Russia; 2 Medical and Genetics Scientific Centre, Russian Academy of Medical Sciences, Moscow, Russia; 3 Institute of Arts, Ethnography and Folklore, National Academy of Sciences of Belarus, Minsk, Belarus

## Abstract

**Background:**

It was demonstrated previously that the three-locus RFLP haplotype, TaqI B-TaqI D-TaqI A (B-D-A), at the DRD2 locus constitutes a powerful genetic marker and probably reflects the most ancient dispersal of anatomically modern humans.

**Results:**

We investigated TaqI B, BclI, MboI, TaqI D, and TaqI A RFLPs in 17 contemporary populations of the East European Plain and Siberia. Most of these populations belong to the Indo-European or Uralic language families. We identified three common haplotypes, which occurred in more than 90% of chromosomes investigated. The frequencies of the haplotypes differed according to linguistic and geographical affiliation.

**Conclusion:**

Populations in the northwestern (Byelorussians from Mjadel'), northern (Russians from Mezen' and Oshevensk), and eastern (Russians from Puchezh) parts of the East European Plain had relatively high frequencies of haplotype B2-D2-A2, which may reflect admixture with Uralic-speaking populations that inhabited all of these regions in the Early Middle Ages.

## Background

The DRD2 gene is located on chromosome 11 and encodes the neuronal dopamine receptor D2, which plays a role in movement, emotional memory, and appetitive behavior [1]. The DRD2 locus was an object of numerous genetic association studies [2-5], and the most extensively studied polymorphism is a TaqI A RFLP (rs1800497; in the vicinity of the DRD2 gene), which has been associated with the pathology of psychoses (schizophrenia and manic-depressive disorder), Parkinson's disease, and various substance abuse syndromes. It has been proposed that TaqI A might be in linkage disequilibrium with some unidentified polymorphisms within the exons or regulatory regions of the DRD2 gene, but recently it has been mapped to the last exon of the ANKK1 (ankyrin repeat and kinase domain containing 1) gene, and it results in a Glu-Lys substitution [6]. Other frequently studied RFLPs, for example, TaqI B and D (rs1079597 and rs1800498, respectively) are located in the introns of the DRD2 gene and, most probably, have no functional significance.

TaqI B, TaqI D, and TaqI A polymorphisms have also been studied on a worldwide scale [[7-11]; the ALFRED database, ], and centers of dispersal, which probably reflect the most ancient dispersal of anatomically modern humans, have been proposed for their three-locus haplotypes [7]. It has been shown that the B2, D2, and A1 alleles are ancestral alleles common to other hominoids [12-14]. Kidd et al. [7] proposed the following evolutionary sequence for the most common haplotypes: evolution of B2-D2-A1 to B2-D2-A2 and B1-D2-A1 and evolution of B2-D2-A2 to B2-D1-A2. The other less frequent haplotypes probably arose by recombination. Three-locus haplotypes exhibit pronounced geographical differentiation. With the exception of some tribal populations of India [11], the ancestral haplotype B2-D2-A1 is mainly confined to African groups. The singly derived haplotype B1-D2-A1 is most widespread among people of East Asian descent, including Native Americans. Haplotype B2-D2-A2 is present among all populations but is least prevalent in Western European and American populations. The doubly derived haplotype B2-D1-A2 is common in Europe and rare in East Asia. The other haplotypes are extremely rare and have sporadic distribution.

Here we provide data on the variability of DRD2 haplotypes in a previously uninvestigated region, the East European Plain. We investigated 14 contemporary populations of the East European Plain and Siberia that belong to the Indo-European and Uralic language families. In addition, two populations of the Altaic language family (Yakuts and Kalmyks) and a population of the North Caucasian language family (Adygeis) were included as reference groups. We also performed an updated global analysis of B-D-A haplotype frequencies using our data and data from the ALFRED database.

## Results

**Figure 1:**
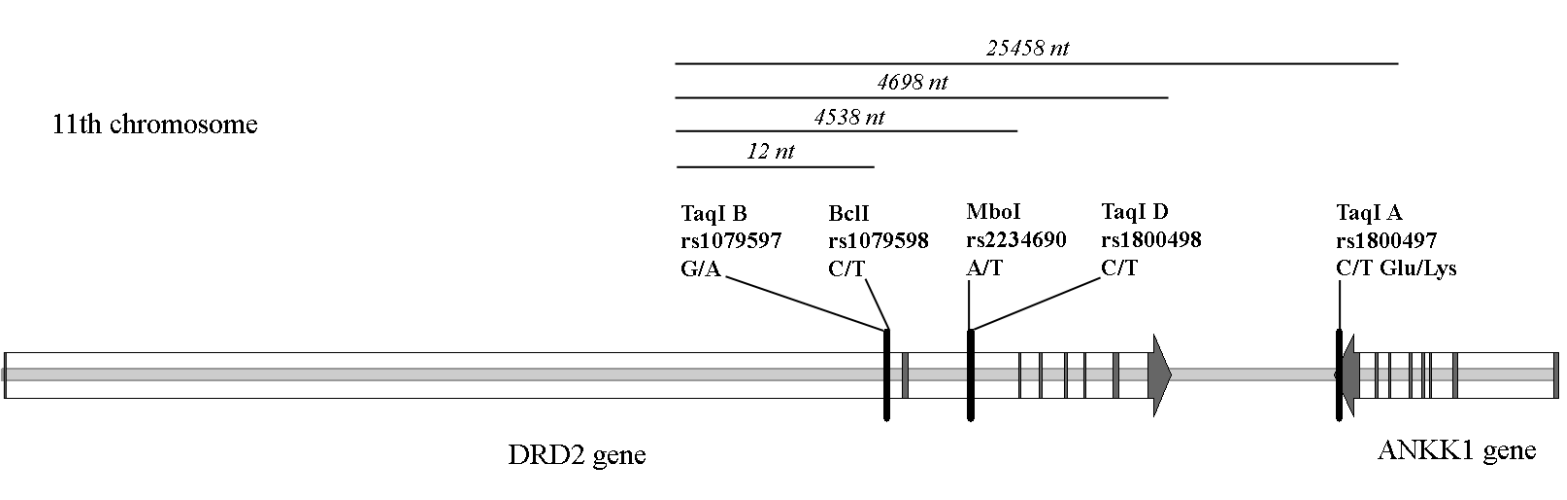
**Map of polymorphisms in the DRD2 and ANKK1 genes**. Restriction polymorphisms, corresponding SNPs, and distances between them are shown. Exons are indicated by gray vertical bars.

We studied five RFLP loci, TaqI B, BclI, MboI, TaqI D, and TaqI A. The locations of these polymorphisms are shown on the gene map (Figure 1), and allele frequencies in the populations studied are presented in Table 1. Allele 1 (the restriction site was absent) at the TaqI B locus was always present with allele 1 at the BclI locus; allele 2 (the restriction site was present) at the former locus was always found with allele 2 at the latter locus. The same tight linkage was observed for the MboI and TaqI D loci, and there were no exceptions among the 3198 chromosomes studied. In most populations, all loci exhibited Hardy-Weinberg equilibrium (assessed by the exact test using a Markov chain, P > 0.05). Significant deviations from Hardy-Weinberg equilibrium were observed only for the MboI and TaqI D loci in Khants (P = 0.044) and the TaqI A locus in Yakuts (P = 0.023).

**Table 1:** Geographical and linguistic affiliations of populations sampled in this study, and allele frequencies in the studied populations

			**SNP No.**	**rs1079597**	**rs1079598**	**rs2234690**	**rs1800498**	**rs1800497**
			
			**SNP allele**	**A^a^**	**T^b^**	**T^c^**	**C^d^**	**T^e^**
			
			**RFLP**	**TaqI B**	**BclI**	**MboI**	**TaqI D**	**TaqI A**
**Language family**	**Population**	**Geographical region**	**Number of individuals**	**allele 1**	**allele 1**	**allele 1**	**allele 2**	**allele 1**

Northern Caucasian	Adygeis (Shapsugs)^f^	Russia, Krasnodar region	98	0.117	0.117	0.367	0.367	0.153

Indo-European	Byelorussians 1^g^	Belarus, Brest region, Pinsk	70	0.129	0.129	0.450	0.450	0.143
			
	Byelorussians 2^g^	Belarus, Minsk region, Mjadel'	75	0.113	0.113	0.487	0.487	0.120
			
	Byelorussians 3^g^	Belarus, Mogilev region, Klimovichi	85	0.153	0.153	0.400	0.400	0.165
			
	Russians 1	Russia, Tver region, Andreapol'	109	0.252	0.252	0.463	0.463	0.261
			
	Russians 2^g^	Russia, Smolensk region, Sychevka	117	0.145	0.145	0.440	0.440	0.154
			
	Russians 3^g^	Russia, Kursk region, Ponyri	65	0.131	0.131	0.446	0.446	0.154
			
	Russians 4^h^	Russia, Ivanovo region, Puchezh	95	0.158	0.158	0.526	0.526	0.189
			
	Russians 5^g^	Russia, Archangelsk region, Oshevensk	71	0.211	0.211	0.577	0.577	0.232
			
	Russians 6	Russia, Archangelsk region, Mezen'	147	0.153	0.153	0.554	0.554	0.156

Uralic	Veps	Russia, Vologda region, Babaevo	97	0.196	0.196	0.500	0.500	0.180
			
	Komi 1 (Izhemski)^i^	Russia, Komi Republic, Izhma	112	0.138	0.138	0.411	0.411	0.152
			
	Komi 2 (Priluzski)^i^	Russia, Komi Republic, Obyachevo	109	0.206	0.206	0.482	0.482	0.225
			
	Khants^j^	Russia, Khanty-Mansi autonomous region	62	0.234	0.234	0.750	0.750	0.258
			
	Nenets	Russia, Yamalo-Nenetsky autonomous region	65	0.223	0.223	0.762	0.762	0.262

Altaic	Yakuts^g^	Russia, Saha Republic, Tiungiuliu	118	0.269	0.269	0.932	0.932	0.214
			
	Kalmyks^g^	Russia, Republic Kalmykiya, Elista	104	0.356	0.356	0.856	0.856	0.346

SNP accession numbers and corresponding RFLPs names are presented for the polymorphisms studied. Alleles without restriction sites are named "allele 1", alleles with restriction sites "allele 2".

^a ^only A and G were detected at this SNP locus in a sample of 2450 chromosomes from 17 populations ,

^b ^only T and C were detected at this SNP locus in a sample of 1204 chromosomes from 14 populations

^c ^only A and T were detected at this SNP locus in a sample of 142 chromosomes from three populations

^d ^only T and C were detected at this SNP locus in a sample of 946 chromosomes from 12 populations

^e ^only T and C were detected at this SNP locus in a sample of 2628 chromosomes from 21 populations ,

^f ^population initially described in [62]

^g ^population initially described in [38]

^h ^population initially described in [37]

^i ^population initially described in [53]

^j ^population initially described in [39]

Alleles 1 at the TaqI B, BclI, and TaqI A loci were more frequent in the populations of Russians 1 and 5, Veps, Komi 2, Khants, Nenets, Yakuts, and especially Kalmyks, than in the other populations (Table 1). Allele 1 at the MboI locus and allele 2 at the TaqI D locus were the most frequent alleles in Asian populations, i.e. Khants, Nenets, Yakuts, and Kalmyks. It is notable that combinations of allele 2 at the TaqI B locus with allele 1 at the BclI locus and allele 2 at the MboI locus with allele 2 at the TaqI D locus, found in the sequenced chimpanzee genome [15], were absent in human populations (Table 1).

Pairwise linkage disequilibrium (D') was strong in most cases (Table 2). However, disequilibrium values were lower for the MboI-TaqI A and TaqI D-TaqI A pairs in some populations, which is similar to the findings of Kidd et al. [7]. In Yakuts, disequilibrium was very low, except for the TaqI B-TaqI A and BclI-TaqI A pairs. Only two of the SNPs studied have been involved in the international HapMap project : TaqI A (rs1800497) and TaqI B (rs1079597). Linkage disequilibrium between these SNPs is high and significant in all 11 populations included in the HapMap project phase 3. The MboI and TaqI D loci are located between SNPs rs2587548 and rs2734836 investigated in the HapMap project. Linkage disequilibrium between SNP rs2587548 and the TaqI A locus is low in the Utah population of European ancestry and high but nonsignificant in the Chinese and Japanese populations (HapMap project phase 2), which is in agreement with our results.

**Table 2:** Pairwise linkage disequilibrium values for the studied populations

**Population**	**TaqIB/BclI - MboI/TaqID**	**TaqIB/BclI - TaqIA**	**MboI/TaqID - TaqIA**
Adygeis	1.000 *	1.000 *	0.458 *
Byelorussians 1	1.000 *	1.000 *	0.870 *
Byelorussians 2	1.000 *	1.000 *	0.824 *
Byelorussians 3	1.000 *	0.953 *	0.835 *
Russians 1	1.000 *	1.000 *	0.956 *
Russians 2	1.000 *	1.000 *	1.000 *
Russians 3	1.000 *	1.000 *	0.855 *
Russians 4	1.000 *	1.000 *	0.507 *
Russians 5	1.000 *	1.000 *	0.774 *
Russians 6	1.000 *	1.000 *	0.919 *
Veps	1.000 *	1.000 *	1.000 *
Komi 1	1.000 *	1.000 *	0.930 *
Komi 2	1.000 *	1.000 *	0.833 *
Khants	1.000 *	1.000 *	1.000 *
Nenets	1.000 *	1.000 *	0.498 P = 0.155
Yakuts	0.061 P = 0.827	1.000 *	0.020 P = 0.927
Kalmyks	1.000 *	0.978 *	1.000 *

The modulus of the standardized linkage disequilibrium coefficient (D') of Lewontin [57] is shown. An asterisk indicates significance level P < 0.05; greater P values are written in full.

**Figure 2:**
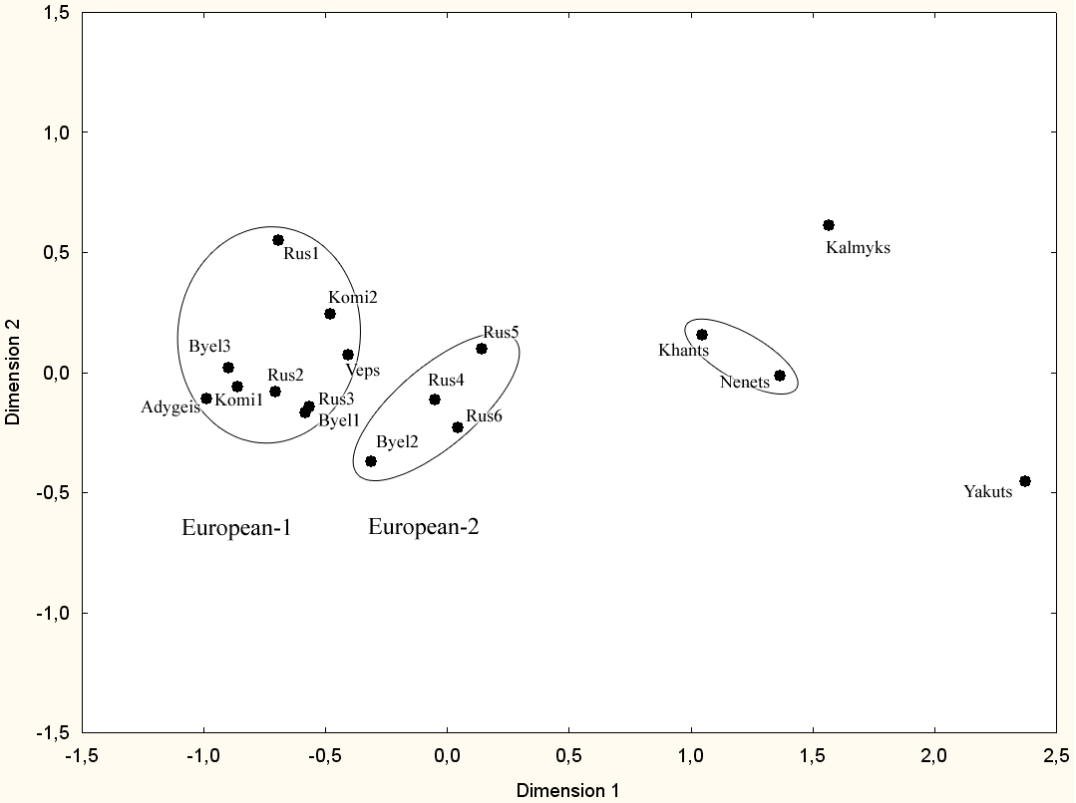
**A two-dimensional scaling plot of F_ST_-based genetic distances between the study populations, Russia and Belarus**. Distances were inferred from haplotype frequencies. Population groups defined by UPGA cluster analysis are indicated by ellipses.

Taking into consideration perfect linkage in the TaqI B-BclI and MboI-TaqI D pairs, the BclI and MboI RFLPs were redundant for inter-population comparison, and we focused on the distribution of three-locus haplotypes TaqI B-TaqI D-TaqI A. Their frequencies are shown in Table 3. Only three haplotypes were common in the populations studied: B2-D1-A2, B2-D2-A2, and B1-D2-A1 (haplotypes termed according to [7]). The other haplotypes were extremely rare. Haplotype frequencies were used for calculation of F_ST_-based genetic distances. The resulting distance matrix was visualized using multidimensional scaling (MDS) (Figure 2). Two large population groups can be distinguished: Asian (Khants, Nenets, Kalmyks, and Yakuts) and European (the other populations). According to UPGA cluster analysis, the European and Asian groups might be further subdivided into subclusters European-1 and European-2, Khants-Nenets group, Kalmyks, and Yakuts.

**Table 3:** Frequencies of TaqI B-TaqI D-TaqI A haplotypes in the study populations

**Population**	**Haplotype**							
	
	**GTC**	**GCC**	**ACT**	**GCT**	**GTT**	**ACC**	**ATC**	**ATT**
	
	**B2-D1-A2**	**B2-D2-A2**	**B1-D2-A1**	**B2-D2-A1**	**B2-D1-A1**	**B1-D2-A2**	**B1-D1-A2**	**B1-D1-A1**
Adygeis 1 (Shapsugs)	0.597	0.250	0.117		0.036			
Byelorussians 1	0.543	0.314	0.129	0.007	0.007			
Byelorussians 2	0.507	0.373	0.113		0.007			
Byelorussians 3	0.582	0.247	0.147		0.018	0.006		
Russians 1	0.529	0.209	0.252	0.002	0.008			
Russians 2	0.560	0.286	0.145	0.009				
Russians 3	0.536	0.311	0.131	0.005	0.018			
Russians 4	0.442	0.368	0.158		0.032			
Russians 5	0.401	0.366	0.211		0.021			
Russians 6	0.442	0.401	0.153		0.003			

Veps	0.500	0.304	0.180			0.015		
Komi 1 (Zyrian)	0.585	0.263	0.138	0.009	0.004			
Komi 2 (Zyrian)	0.505	0.271	0.206	0.005	0.014			

Khants 1	0.250	0.492	0.234					

Nenets	0.222	0.476	0.262		0.040			

Yakuts 1	0.047	0.684	0.197			0.050	0.005	0.016
Kalmyks	0.139	0.500	0.341		0.005	0.014		

In the Asian cluster genetic distances were significant between Yakuts and Kalmyks (P < 0.00001), Khants and Yakuts (P < 0.00001), Khants and Kalmyks (P < 0.04661), Nenets and Yakuts (P < 0.00099), but not between Nenets and Kalmyks (P < 0.07438). The distance between Khants and Nenets was small and non-significant (P < 0.52033).

All populations of the European cluster had significant genetic distances from the populations of the Asian cluster. The European-1 and European-2 clusters were quite homogeneous; most distances were non-significant within the former, and all were non-significant within the latter. Only Russians 1 occupied distinct position within the European-1 cluster. In contrast, most pairwise genetic distances between the two subclusters were significant. Exceptions included Veps, which had no significant distances from the populations of the European-2 cluster, and Byelorussians 2, which, had a small number of significant distances from the populations of the European-1 cluster.

The matrix of haplotype-based genetic distances was also compared with matrices of great circle geographical distances (data not shown) to assess the possible effect of isolation by distance. The matrices including all 17 populations were highly correlated according to the Mantel test (r = 0.749, P-value = 0.0001). However, correlation of geographical and genetic distances was not observed among the populations of the European cluster, i.e., after exclusion of Khants, Nenets, Kalmyks (which have migrated from East Asia in historical times), and Yakuts (r = 0.121, P-value = 0.2999). The use of more realistic distance measurements around geographical barriers, such as the Azov Sea, the Caucasus, and some parts of the Ural Mountains had little effect on results of the test (the correlation coefficients for 17 and 13 populations were 0.754 and 0.117, respectively; P-values were 0.0001 and 0.3109, respectively). Thus, isolation by distance is not a likely cause of the genetic variation observed in the East European Plain.

**Figure 3:**
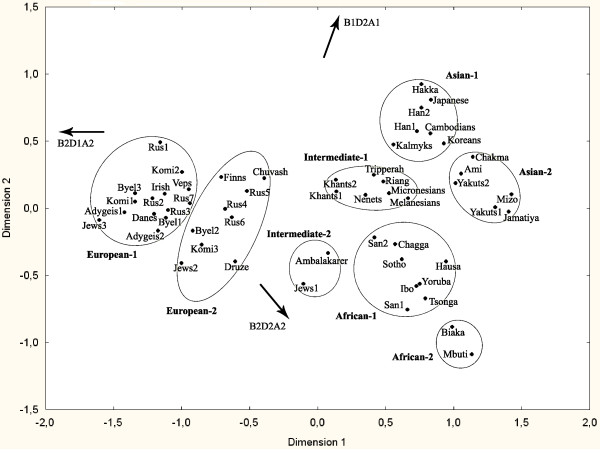
**A two-dimensional scaling plot of F_ST_-based genetic distances between the study and the reference populations**. Gradients of haplotype frequencies for the three most common haplotypes are indicated by arrows. Population groups identified by cluster analysis are highlighted by ellipses.

We also compared our results with those of previous authors to examine population relationships in greater detail. To do this, we analyzed haplotype frequencies in the populations studied and in 38 populations from various continents (data from [7,9], and the ALFRED database) using MDS of F_ST_-based genetic distances (Figure 3 and Additional file 1). A good fit between the two-dimensional plot and the source data was obtained, demonstrated by the low stress value (0.062). Cluster analysis by the UPGA algorithm was performed using the same distance matrix and enabled us to define four large population clusters with two subclusters each, i.e., eight population groups in total. All clustering methods that we used (complete linkage, unweighted and weighted pair-group average, unweighted and weighted pair-group centroid, and Ward's method) revealed identical clusters at the level of eight groups. However at 'higher' and 'lower' clustering levels, some methods gave different results. To identify haplotypes responsible for the observed intercluster differences, haplotype frequencies for various clusters were compared using the Kolmogorov-Smirnov test (Table 4).

**Table 4:** Comparison of some population groups according to the Kolmogorov-Smirnov test of haplotype frequencies

	**B2-D1-A2**	**B2-D2-A2**	**B1-D2-A1**
European-1/European-2	0.0010	< 0.0001	**0.4774**

European-2/Intermediate-1	0.0002	0.0002	0.0075

Asian-1/Asian-2	0.0385	0.0012	0.0082

Intermediate-1/Asian-1	0.0082	**0.9441**	0.0006

Intermediate-1/Asian-2	0.0012	0.0082	**0.3328**

P-values are shown; those highlighted in bold indicate that the frequency distributions of the two samples are not significantly different (P > 0.05).

The following populations constituted the European-1 subcluster: Indo-European-speaking Irish, Danes, Byelorussians 1 and 3, Russians 1, 2, 3, and 7; North-Caucasian-speaking Adygeis 1 and 2; Jews 3 (Ashkenazi); Uralic-speaking Veps, Komi 1 and 2. The populations of this cluster had the following haplotype frequencies: 0.50-0.65 for the "European" haplotype B2-D1-A2, 0.21-0.31 for the "worldwide" haplotype B2-D2-A2, 0.08-0.25 for the "East Asian" haplotype B1-D2-A1. Some populations of the East European Plain (Indo-European-speaking Byelorussians 2, and Russians 4, 5, and 6; Uralic-speaking Finns and Komi 3; Altaic-speaking Chuvash) fell into the European-2 subcluster, which is characterized by the following haplotype frequencies: B2-D1-A2, 0.37-0.52; B2-D2-A2, 0.27-0.45; and B1-D2-A1, 0.04-0.25. Two European subclusters can be differentiated on the basis of their B2-D2-A2 and B2-D1-A2 frequencies (P-values according to the Kolmogorov-Smirnov test < 0.0001 and 0.001, respectively). This is also evident from Figure 3: on the MDS plot, both European subclusters occupy almost identical positions in the B1-D2-A1 frequency gradient (Figure 3, upward arrow) but have different positions in the B2-D1-A2 and B2-D2-A2 frequency gradients (Figure 3, arrows).

A low but highly significant level of population differentiation was observed between the European-1 and -2 subclusters (F_CT _0.018, P < 0.00001, Table 5); 59% of genetic distances between populations of the two subclusters were significant, but only 14% and 22% of the intracluster distances were significant. Other close pairs of subclusters such as Intermediate-1 and -2 demonstrated higher levels of differentiation (F_CT _0.027-0.045, Table 5). About 60% of intercluster genetic distances were significant in all instances.

**Table 5:** Indexes of population structure

**Standardized variance**	**No. of samples**	**F_ST_**
Among populations:		
Global analysis	55	0.114****
European	23	0.013****
European-1	14	0.003*
European-2	9	0.006*
Intermediate	9	0.009*
African	10	0.019***
Asian	13	0.026****

		F_CT_

Among groups:		
European-Intermediate-Asian-African		0.143****
European-Intermediate		0.099****
European-African		0.179****
European-Asian		0.199****
Intermediate-African		0.042****
Intermediate-Asian		0.028****
African-Asian		0.059****
European-1-European-2		0.018****
European-2-Intermediate-1-Intermediate-2		0.058****
European-2-Intermediate-1		0.065**
European-2-Intermediate-2		0.040*
European-2-Asian-1		0.143***
Intermediate-1-Intermediate-2		0.027*
Intermediate-1-African-1		0.040**
Intermediate-1-Asian-1-Asian-2		0.036****
Intermediate-1-Asian-1		0.025**
Intermediate-1-Asian-2		0.041**
African-1-African-2		0.031*
Asian-1-Asian-2		0.045**

* P < 0.05

** P < 0.001

*** P < 0.0001

**** P < 0.00001

Ugric-speaking Khants 1 and 2 and Samoyedic-speaking Nenets were distant from the other Uralic populations (Finns, Veps, Komi 1, 2, and 3) and grouped into the Intermediate-1 subcluster with Sino-Tibetan-speaking Riang and Tripperah from India, with Melanesians and Micronesians (the results for Melanesians and Micronesians should be interpreted with caution because of a low sample size; see Additional file 1). In Khants and Nenets, the frequencies of the "European" haplotype B2-D1-A2 (0.22-0.25) and the "East Asian" haplotype B1-D2-A1 (0.23-0.26) were intermediate between those of the European-1 and Asian-1 subclusters (Figure 3). It is interesting that the frequencies of the "worldwide" haplotype B2-D2-A2 in Khants (0.48-0.49) and Nenets (0.48) fell within the range typical of the Asian-1 subcluster, 0.44-0.53 (P-value = 0.9441 according to the Kolmogorov-Smirnov test).

The Asian-1 subcluster was formed by populations of East Asian descent (Altaic-speaking Kalmyks; Koreans and Japanese; Sino-Tibetan-speaking Hakka, Han 1 and 2; and Austro-Asiatic-speaking Cambodians). They had the following frequency pattern: B2-D1-A2, 0.04-0.14; B2-D2-A2, 0.44-0.53; and B1-D2-A1, 0.34-0.46.

The global F_ST _value, 0.11413 (Table 5), falls within the range typical of autosomal markers (0.09-0.14, [16]). This value was estimated using haplotype frequencies without taking into account the extent of molecular differences between haplotypes. Calculation of F_ST _based on numbers of pairwise differences between haplotypes gave a nearly identical value, 0.11383. Thus, the differentiation of populations may be explained by drift only, without any significant influence of mutation.

## Discussion

The East European (Russian) Plain is a region in which peoples of the Indo-European and Uralic language families have come into contact over an extended period. Uralic-speaking peoples have the longest validated archaeological record in this region [17]. The most recent large-scale migration to this region involved the movement of Slavs (the Indo-European language family) to the east and northeast of their presumed homeland in Central Europe about 500 AD [18,19]. Slavs were not the first Indo-European-speaking people who arrived in the Russian Plain: in the first millennium BC, Baltic-speaking tribes occupied a large part of the East European Plain [17]. They were later displaced by Slavic tribes. According to the widely accepted hybridization theory of the origin of Eastern Slavs [20], Slavic populations arriving in the East European Plain were mixed with indigenous Uralic- and, probably, Baltic-speaking people.

In our study, all populations of the East European Plain (excluding the Kalmyks, which are of East Asian origin) fell into a single large cluster termed European. Many populations within this cluster are indistinguishable with our genetic marker, i.e., genetic distances between them were not significant, which is in agreement with the low F_ST _value for the European cluster (0.013). However, some populations were characterized by a large percentage of significant genetic distances from the other populations of the cluster. Most such populations fell into the so-called European-2 subcluster defined by cluster analysis; the 'core' subcluster was termed European-1, and 59% of genetic distances between populations of the two subclusters were significant. European-1 and European-2 subclusters (Figure 3) are differentiated according to the B2-D2-A2 frequency, but not according to the B1-D2-A1 frequency, which might reflect the degree of Asian admixture. Natural selection probably was not responsible for separation of the two European subclusters as there is no difference in allele frequencies at the TaqI A locus (Table 4), which is considered the most likely candidate for selection in the whole DRD2 region [2-6].

The European-2 subcluster includes two Middle Eastern populations (Jews 2 from Yemen and Druze from Israel), two Uralic-speaking populations (Finns and Komi 3), also four Slavic-speaking populations (Byelorussians 2 and Russians 4, 5, and 6), and the Altaic-speaking Chuvash. All these linguistically and geographically distant populations are differentiated to some extent from the core of the European cluster, the European-1 subcluster, because of a relatively high B2-D2-A2 frequency.

The B2-D1-A2 and B1-D2-A1 haplotypes apparently have centers of dispersal in Europe/West Asia and East Asia, respectively [7]. The B2-D2-A2 haplotype may also have a center of dispersal, the most probable location of which is in Africa. B2-D2-A2 was among the first haplotypes that evolved from the ancestral haplotype B2-D2-A1 in Africa [7] and still is the most abundant haplotype in all African populations (Additional file 1). Therefore, the first settlers of Eurasia that migrated to Arabia and Levant may mostly have carried the B2-D2-A2 haplotype and a small proportion of other haplotypes that were either subsequently eliminated or amplified by genetic drift and/or natural selection in various parts of the world.

B2-D2-A2 is the predominant haplotype in contemporary populations of Middle Eastern origin (Jews 1 from Ethiopia, Jews 2 from Yemen, and Druze from Israel). Populations of Levant began to disperse into Europe about 50,000 YBP [21,22]. People of that diaspora might have carried B2-D2-A2 as a prevalent haplotype. However, another haplotype, B2-D1-A2, is the predominant haplotype in contemporary populations of Europe, for example, in linguistically and geographically distant populations such as the Irish, Danes, Russians, and Adygeis. Amplification of this haplotype might have taken place during the initial migration to Europe or might be associated with confinement in refugia of the last glacial maximum and reexpansion. According to one of several hypotheses based on archaeological evidence (reviewed by Zvelebil [23]), Uralic-speaking groups descended from Europeans who had been confined in the East European refugium in Ukraine and Southern Russia [24-29]. It is possible that the people of the East European refugium retained a high frequency of the B2-D2-A2 haplotype, in contrast with the other European populations that have spread from other refugia.

Thus, on a global scale, the B2-D2-A2 frequency possibly reflects genetic features of the first Eurasian settlers, e.g. in Jews 2 from Yemen and Druze from Israel, and, on a local scale, genetic features of Uralic-speaking populations, e.g., in Finns and Komi 3, Khants and Nenets. Therefore, the other members of the European-2 subcluster, Byelorussians 2, Russians 4, 5, and 6, and Chuvash, probably have a certain level of Uralic admixture. Moreover, according to other genetic and historical data, these populations have Uralic substratum.

Studies on mtDNA [30-34] and Y-chromosome haplogroups [33,35,36], autosomal VNTR diversity [37,38], and autosomal haplotypes [39] consistently show a degree of Uralic admixture in populations of northern Russians. This admixture is manifested by a 'Uralic-specific' marker, the U4 mtDNA haplogroup [40-45], or by Asian-specific markers which Uralic populations acquired during earlier admixture with East Asians: Asian mtDNA haplogroups [40,42,45], N3 and N2 Y-chromosome haplogroups [46,47]. For example, the Mezen' population investigated by Balanovsky et al. [36] has high N3 frequency, resembling some Uralic populations.

Russians 5 (Oshevensk) were most closely associated with Finns and Chuvash according to the MDS results (Figure 3). In the study of Verbenko et al. [38] on polymorphic tandem repeats at the D1S80 locus, the same Oshevensk sample (as well as another northern Russian sample) clustered together with Uralic-speaking Mari, Komi, and Udmurts, whereas other Russian populations clustered with Indo-Europeans and Adygeis. Analysis of other repeat loci, 3' ApoB, DMPK, DRPLA, and SCA1, also demonstrated remoteness of some northern Russian populations (including Russians 5, Oshevensk) from the core of the European cluster [38]. Similar results were obtained using haplotypes at the TP53 locus [39]: the Oshevensk population tended to form a cluster with Uralic-speaking Mordvins and with Altaic-speaking Kalmyks and Buryats, but not with Russians from Smolensk (Russians 2) or Byelorussians from Pinsk (Byelorussians 1).

**Figure 4:**
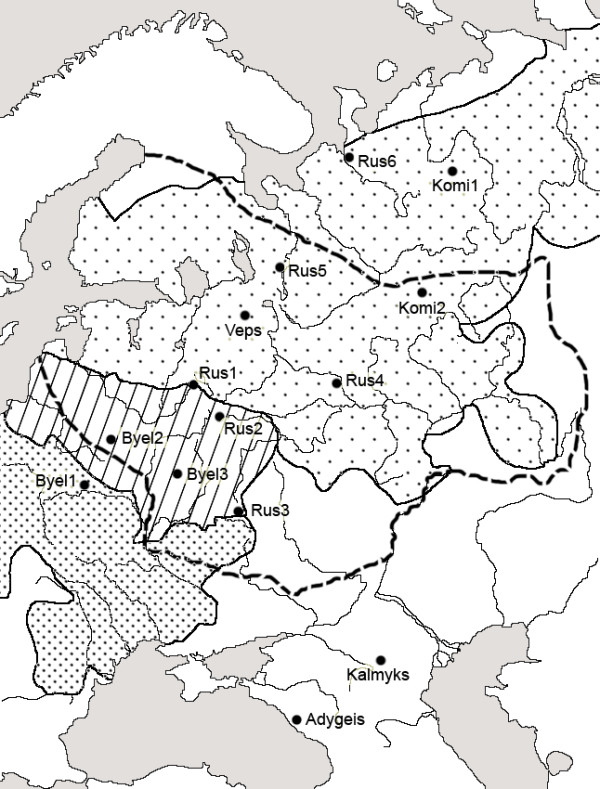
**A map of archaeological cultures on the East European Plain showing the locations of the study populations**. The dashed line indicates the region that was presumably occupied by Uralic-speaking peoples in the Early Neolithic era, the 5^th ^millennium BC (according to [17]). Solid lines indicate three regions occupied by different ethnic groups in the 1^st^-6^th ^centuries AD: Slavic- and Germanic-speaking tribes, densely dotted area; Baltic-speaking tribes, hatched area; Uralic-speaking tribes, sparsely dotted area [48,61].

According to archaeological data, the Arkhangelsk region (including Mezen' and Oshevensk) was populated by Uralic tribes in the Middle Ages ([48]; see Figure 4). Russian colonization of this region began relatively recently (after the 12th century AD) [48]. Thus, a very high level of Uralic admixture in Mezen' and Oshevensk is not surprising.

The Uralic genetic substratum is appreciable not only in the Northeast of the East European Plain but also in its northwestern part, for example, in the Pskov [33] and Novgorod regions [38], in Latvians and Lithuanians [46,49,50]. Baltic-speaking peoples, now represented by the Latvians and Lithuanians, came into contact with Uralic groups before the Slavs did (Figure 4; [18]). That Byelorussians 2 (Mjadel') fell into the European-2 subcluster may also reflect a general tendency in the northwestern region. Mjadel' is located in the northwestern part of Belarus near the contemporary Lithuanian border. The Russians 4 (Puchezh) population is distant from the northeastern and northwestern groups, but also belongs to the European-2 subcluster (Figure 3). Uralic admixture in this population may be explained by the presence of Uralic-speaking tribes in the region of Puchezh in historical times ([51]; see Figure 4).

Russians 1 (Andreapol') and Uralic-speaking Veps are close to Uralic-speaking Komi 2 (Obyachevo) on the MDS plot (Figure 3). All these populations are located within the region occupied by Uralic peoples in the Middle Ages (Figure 4), but belong to the European-1 cluster and do not have high B2-D2-A2 frequencies typical of the European-2 cluster. However, they are shifted from the core of the European cluster because of a relatively high proportion of the "East Asian" haplotype B1-D2-A1. In fact, the Veps population has significant genetic distance only from Druze, but not from the other populations of the European-2 cluster, and Komi 2 only from Jews 2, Druze, Russians 6, and Komi 3. The Andreapol' sample had the highest B1-D2-A1 frequency of all European populations (Additional file 1), and eight of 13 genetic distances between this sample and the other populations of the European-1 subcluster are significant.

Komi populations demonstrate remarkable heterogeneity according to various marker systems. For example, Komi-Permyaks and Komi-Zyrians have rather different mtDNA haplogroup frequencies but both have a relatively high U4 frequency [40]. In our study, one of the Komi-Zyrian populations (Komi 1, Izhma) belonged to the core of the European-1 cluster. It is interesting that the craniological results of Moiseyev [52] also place Komi-Zyrians at the core of the European cluster and distant from Uralic and Asian groups. According to three-site haplotype frequencies at the TP53 locus and VNTR frequencies at the D1S80 and 3' ApoB loci, the Komi 1 and 2 populations are distant from Uralic-speaking Finns, Mordvins and Khants, East Asian groups, and Slavic groups [53]. Moreover, the Komi 2 (Obyachevo) population is distant from Komi 1 and closer to Slavic groups than Komi 1 [53]. Thus, the position of Komi in genetic gradients remains uncertain because of substantial divergence of population samples and contradictory results, which may reflect a complex history of this group or natural selection.

B2-D2-A2 haplotype frequencies in Khants (0.48-0.49) and Nenets (0.48) fell within the range typical of the Asian-1 subcluster, 0.44-0.53. Therefore, the high frequency of the B2-D2-A2 haplotype in Uralic-speaking Khants and Nenets cannot be explained only by admixture of Europeans with typical East Asians; a combination of admixture and other processes such as gene drift or selection is a more likely explanation. The Khants and Nenets may have received the B2-D2-A2 haplotype both from the East European refugium and from East Asia, e.g., from Siberian populations of East Asian origin. One of such Siberian populations, the Yakuts, has a very high frequency of haplotype B2-D2-A2, 0.6-0.7. However, DRD2 haplotype frequencies in other Siberian populations of East Asian origin are unknown, and it is not possible to draw definitive conclusions about B2-D2-A2 frequencies in Siberia based on one ethnic group alone. Moreover, because Yakuts 1 exhibited low linkage disequilibrium between RFLPs, haplotype frequencies for this sample should be interpreted with caution. Yakuts 1 and 2 apparently do not belong to the cluster of typical East Asians, East Asian-1 (Figure 3), although they are clearly of East Asian origin. As suggested by archaeological and linguistic evidence, the Yakuts probably migrated north from their original area of settlement near Lake Baykal because of the Mongol expansion from the 13th to 15th century AD [54]. Y-chromosome results reveal a very strong bottleneck in the Yakut population, which probably preceded their recent expansion [46,54]. This bottleneck effect may be responsible for the aberrant haplotype frequencies for Yakuts observed in our study.

## Conclusion

Populations in the northwestern (Byelorussians 2 from Mjadel'), northern (Russians 5 from Mezen' and 6 from Oshevensk; Komi 3), and eastern parts (Russians 4 from Puchezh and Chuvash) of the East European Plain have relatively high frequencies of haplotype B2-D2-A2, which may reflect admixture with Uralic-speaking populations. Uralic genetic substratum in these regions, which were inhabited by Uralic-speaking tribes as late as the Early Middle Ages, was also shown by studies in which other genetic markers were used (mtDNA, Y-chromosome, and autosomal). Thus, the analysis of DRD2 haplotypes supports results on Slavic-Uralic admixture obtained using other markers, mainly neutral and sex-specific markers.

## Methods

### Populations

**Figure 5:**
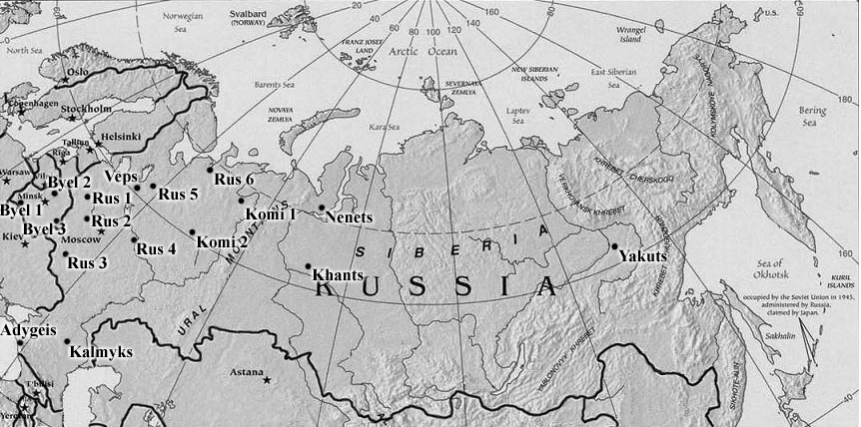
**Geographical location of the study populations on the map of Russia and the neighboring countries**.

The linguistic affiliations and geographical locations of the studied populations are shown in Table 1 and Figure 5. These populations have been described previously (see footnotes to Table 1), except for Andreapol' (Russians 1), Mezen' (Russians 6), Veps, and Nenets. The Andreapol' sample from the Tver region, the most western of the Russian populations involved in our study, consisted of individuals born in Andreapol' (a small town), Bologovo village, and other nearby villages. The Mezen' sample from the Arkhangelsk region, the most northern of the Russian populations sampled, consisted of individuals born in the small towns, Mezen' and Kamenka, in Dorogorskoe village, and in other nearby villages. The Nenets are Samoyedic-speaking tribes, and their economy is based on reindeer herding. In 2002, the population of Nenets was about 41,000. The Nenets sample, which belongs to the Tundra group, was collected in Yar-Sale and Panaevka villages in the Yamalo-Nenets autonomous district, Tyumen' region. The Veps are Finnic-speaking and reside in small settlements scattered among Russians of the Vologda region, the Leningrad region, and Karelia. In 2002, the population of Veps was about 8,000. Samples were collected from Veps in several villages (Pyazhozero, Pyazhelka, Koloshma, and others) in the Babaevo district of the Vologda region.

### DNA isolation and typing

Blood samples (8 ml) were obtained by venipuncture and collected into EDTA-coated containers. Informed consent was obtained from each individual. The research protocols and forms of informed consent have been approved by the Ethic Commission of the Medico-Genetic Scientific Centre of the Russian Academy of Medical Sciences (an approval was signed by the Head of the Ethic Commission, PhD, professor L.F. Kurilo). All individuals belonged to the native ethnic group of the region, i.e., their lineage in the region extended for at least two previous generations, and were unrelated to each other. DNA was isolated from leucocytes by proteinase K treatment and extraction with phenol-chloroform [55]. Each DNA sample was subjected to three PCR analyses: amplification of a 459 bp fragment for TaqI B and BclI RFLP analysis, amplification of a 300 bp fragment for MboI and TaqI D RFLP analysis, and amplification of a 237 bp fragment for TaqI A RFLP analysis (primer sequences and original PCR protocols were obtained from the website of K. Kidd: ). The locations of these polymorphisms are shown on the gene map (Figure 1). All endonuclease restriction reactions were carried out overnight. Samples containing unrestricted fragments were tested at least twice. Restriction products were separated by electrophoresis using a 2.5% agarose gel.

### Statistical analyses

The general strategy of statistical analysis was similar to that used in the work of Poloni et al. [56]. Allele frequencies, correspondence to Hardy-Weinberg equilibrium, and significance of linkage disequilibrium (P-values) were evaluated using Arlequin version 2.0 software . Linkage disequilibrium values (D') between polymorphic loci were calculated as suggested by Lewontin [57]. Frequencies of haplotypes were estimated from RFLP genotype data using the expectation-maximization algorithm of Excoffier and Slatkin [58] implemented in Arlequin 2.0. Genetic affinities among populations were evaluated using coancestry coefficients, or linearized pairwise F_ST _values [59] calculated on the basis of allele or haplotype frequencies. Significance of genetic distances was tested using permutations [60].

Correlation of geographical and genetic distances was assessed using the Mantel test and XLSTAT version 2008.6.04 software (Addinsoft). Great circle geographical distances were calculated using the haversine formula; latitudes and longitudes were determined using Google Earth software. Multidimensional scaling (MDS) and cluster analysis with the unweighted pair-group average (UPGA) method were performed using STATISTICA version 6.0 . Statistical comparison of haplotype frequencies in population groups was performed using the Kolmogorov-Smirnov test implemented in XLSTAT. Differentiation between population groups defined by MDS and cluster analysis was estimated using the analysis-of-molecular-variance approach, AMOVA [60], implemented in Arlequin 2.0. Conventional F_ST _distances between haplotypes were used, i.e., all haplotypes were considered equidistant (a conservative scenario of pure drift). Significance of the genetic-structure indexes obtained with the AMOVA method was tested using a permutational procedure (1 × 10^6 ^permutations).

## Authors' contributions

OVF carried out the polymorphism typing, performed the statistical analysis and drafted the manuscript. OIL participated in the polymorphism typing. AVK participated in the study design and helped with the statistical analysis and manuscript drafting. LAT, VAS, and AIM carried out sample collection and participated in the study design. SAL conceived of the study, participated in its coordination and helped to draft the manuscript. All authors read and approved the final manuscript.

## Supplementary Material

Additional file 1 **Frequencies of TaqI B-TaqI D-TaqI A haplotypes in the study populations and in reference populations**. This is an extended variant of Table 3 including haplotype frequencies for all populations represented in Figure 3. Click here for file
