# Supplementary material for: Haplotype frequencies at the DRD2 locus in populations of the East European Plain
Source: BMC Genet. 2009 Sep 30;10:62. doi: 10.1186/1471-2156-10-62 (PMC2765450; doi:10.1186/1471-2156-10-62)
Supplement: Additional file 1 — Frequencies of TaqI B-TaqI D-TaqI A haplotypes in the study populations and in reference populations. This is an extended variant of Table 3 including haplotype frequencies for all populations represented in Figure 3. [file 1471-2156-10-62-S1.doc]

## Additional table 1 - Frequencies of TaqI B-TaqI D-TaqI A haplotypes in the study populations and in reference populations

| Language  family ^d^ | Language group ^d^ | Population | Geographical  region | Number of individuals | Haplotype | | | | | | | |
| --- | --- | --- | --- | --- | --- | --- | --- | --- | --- | --- | --- | --- |
|  |  |  |  |  | GCC | GTC | ATT | GTT | GCT | ATC | ACC | ACT |
|  |  |  |  |  | B2-D1-A2 | B2-D2-A2 | B1-D2-A1 | B2-D2-A1 | B2-D1-A1 | B1-D2-A2 | B1-D1-A2 | B1-D1-A1 |
| Khoisan |  | San 1 (Vasekele) ^a^ | South Africa | 50 | 0.081 | 0.522 | 0.053 | 0.168 | 0.129 | 0.047 |  |  |
|  |  | San 2 (Central Kalahari) ^a^ | Botswana, West/Central | 49 | 0.147 | 0.431 | 0.189 | 0.162 | 0.036 | 0.024 |  | 0.011 |
| Niger-Congo |  | Biaka ^a^ | Central African Republic, Southwest, Bagandu | 69 | 0.070 | 0.653 | 0.031 | 0.162 | 0.057 | 0.010 |  | 0.018 |
|  |  | Tsonga ^a^ | South Africa, Limpopo River Valley | 50 | 0.066 | 0.544 | 0.091 | 0.225 | 0.065 |  |  | 0.009 |
|  |  | Ibo ^b^ | Nigeria, Southeast, Enugu | 47 | 0.084 | 0.533 | 0.106 | 0.201 | 0.076 |  |  |  |
|  |  | Yoruba ^b^ | Nigeria, Benin City, | 78 | 0.054 | 0.464 | 0.146 | 0.229 | 0.099 | 0.008 |  |  |
|  |  | Chagga ^b^ | Tanzania, Dar-es-Salaam | 45 | 0.115 | 0.462 | 0.189 | 0.169 | 0.065 |  |  |  |
|  |  | Sotho North ^a^ | South Africa, Northern Province | 50 | 0.1 | 0.42 | 0.23 | 0.25 |  |  |  |  |
| Nilo-Saharan |  | Mbuti ^a^ | Democratic Republic of Congo, Northeast, Ituri Forest | 39 | 0.015 | 0.664 | 0,000 | 0.182 | 0.139 | 0,000 | 0,000 | 0,000 |
| Afro-Asiatic |  | Hausa ^b^ | Nigeria, North Central, Zaria | 38 |  | 0.499 | 0.144 | 0.146 | 0.079 | 0.106 | 0.026 |  |
|  |  | Druze ^a^ | Israel | 74 | 0.432 | 0.453 | 0.068 |  | 0.048 |  |  |  |
|  |  | Jews 1 (Ethiopian) ^a^ | Ethiopia, northwestern (collected in Israel) | 32 | 0.327 | 0.578 | 0.062 | 0,000 | 0.016 | 0.017 |  |  |
|  |  | Jews 2 ^a^ | Yemen (collected in Israel) | 43 | 0.523 | 0.383 | 0.04 | 0.042 |  | 0.013 |  |  |
| North Caucasian |  | Adygeis 1 (Shapsugs) | Russia, Krasnodar region | 98 | 0.597 | 0.250 | 0.117 |  | 0.036 |  |  |  |
|  |  | Adygeis 2 ^a^ | Russia, Krasnodar region | 54 | 0.541 | 0.290 | 0.076 | 0.013 | 0.044 | 0.010 |  | 0.026 |
| Indo-European | Germanic | Jews3 (Ashkenazi) ^b^ | Europe (collected in Israel) | 69 | 0.648 | 0.243 | 0.08 |  | 0.029 |  |  |  |
|  | Celtic | Irish ^b^ | Ireland, northern and central | 116 | 0.519 | 0.252 | 0.132 |  | 0.084 | 0.009 | 0.005 |  |
|  | Germanic | Danes ^a^ | Denmark | 51 | 0.558 | 0.275 | 0.115 |  | 0.031 | 0.022 |  |  |
|  | Slavic | Byelorussians 1 | Belarus, Brest region,  Pinsk | 70 | 0.543 | 0.314 | 0.129 | 0.007 | 0.007 |  |  |  |
|  |  | Byelorussians 2 | Belarus, Minsk region, Mjadel’ | 75 | 0.507 | 0.373 | 0.113 |  | 0.007 |  |  |  |
|  |  | Byelorussians 3 | Belarus, Mogilev region, Klimovichi | 85 | 0.582 | 0.247 | 0.147 |  | 0.018 | 0.006 |  |  |
|  |  | Russians 1 | Russia, Tver region, Andreapol’ | 109 | 0.529 | 0.209 | 0.252 | 0.002 | 0.008 |  |  |  |
|  |  | Russians 2 | Russia, Smolensk region, Sychevka | 117 | 0.560 | 0.286 | 0.145 | 0.009 |  |  |  |  |
|  |  | Russians 3 | Russia, Kursk region,  Ponyri | 65 | 0.536 | 0.311 | 0.131 | 0.005 | 0.018 |  |  |  |
|  |  | Russians 4 | Russia, Ivanovo region, Puchezh | 95 | 0.442 | 0.368 | 0.158 |  | 0.032 |  |  |  |
|  |  | Russians 5 | Russia, Archangelsk region, Oshevensk | 71 | 0.401 | 0.366 | 0.211 |  | 0.021 |  |  |  |
|  |  | Russians 6 | Russia, Archangelsk region, Mezen’ | 147 | 0.442 | 0.401 | 0.153 |  | 0.003 |  |  |  |
|  |  | Russians 7 ^b^ | Russia, Vologda region | 48 | 0.500 | 0.313 | 0.156 |  | 0.021 | 0.011 |  |  |
| Uralic | Finno-Permic | Finns ^a^ | Finland | 36 | 0.420 | 0.274 | 0.194 |  | 0.028 | 0.019 | 0.065 |  |
|  |  | Veps | Russia, Vologda region, Babaevo | 97 | 0.500 | 0.304 | 0.180 |  |  | 0.015 |  |  |
|  |  | Komi 1 (Zyrian) | Russia, Komi Republic,  Izhma | 112 | 0.585 | 0.263 | 0.138 | 0.009 | 0.004 |  |  |  |
|  |  | Komi 2 (Zyrian) | Russia, Komi Republic, Obyachevo | 109 | 0.505 | 0.271 | 0.206 | 0.005 | 0.014 |  |  |  |
|  |  | Komi 3 (Zyrian) ^b^ | Russia, Komi Republic, Kazak | 47 | 0.489 | 0.394 | 0.074 |  | 0.043 |  |  |  |
|  | Ugric | Khants 1 | Russia, Khanty-Mansi autonomous region | 62 | 0.250 | 0.492 | 0.234 |  |  |  |  |  |
|  |  | Khants 2 ^b^ | Russia, Yamalo-Nenetsky autonomous region, Beloyarsk | 49 | 0.245 | 0.478 | 0.265 |  |  |  |  |  |
|  | Samoyedic | Nenets | Russia, Yamalo-Nenetsky autonomous region | 63 | 0.222 | 0.476 | 0.262 |  | 0.040 |  |  |  |
| Altaic |  | Chuvash ^b^ | Russia, Chuvash Republic | 42 | 0.369 | 0.369 | 0.250 |  | 0.012 |  |  |  |
|  |  | Yakuts 1 | Russia, Saha Republic, Tiungiuliu | 118 | 0.047 | 0.684 | 0.197 |  |  | 0.050 | 0.005 | 0.016 |
|  |  | Yakuts 2 ^a^ | Russia, Saha Republic, around Yakutsk | 51 | 0.049 | 0.607 | 0.273 |  |  | 0.050 |  |  |
|  |  | Kalmyks | Russia, Republic Kalmykiya, Elista | 104 | 0.139 | 0.500 | 0.341 |  | 0.005 | 0.014 |  |  |
| isolate (probably Altaic) |  | Koreans ^a^ | Korea | 127 | 0.048 | 0.534 | 0.355 | 0.043 |  | 0.005 | 0.004 | 0.011 |
| isolate (probably Altaic) |  | Japanese ^a^ | Japan (collected in the USA) | 51 | 0.059 | 0.480 | 0.441 | 0.020 |  |  |  |  |
| Sino-Tibetan |  | Han 1 ^a^ | Taiwan, around Taipei | 50 | 0.090 | 0.499 | 0.380 | 0.011 | 0.010 | 0.010 |  |  |
|  |  | Han 2 ^a^ | China (collected in the USA) | 60 | 0.058 | 0.456 | 0.415 | 0.027 |  | 0.035 | 0.008 |  |
|  |  | Hakka ^b^ | Taiwan | 42 | 0.059 | 0.440 | 0.463 | 0.013 |  | 0.025 |  |  |
|  |  | Chakma ^c^ | India, Northeast | 10 |  | 0.65 | 0.35 |  |  |  |  |  |
|  |  | Mizo ^c^ | India, Northeast, Mizoram state | 25 |  | 0.731 | 0.231 | 0.019 |  |  |  | 0.019 |
|  |  | Jamatiya ^c^ | India, Northeast | 55 | 0.036 | 0.718 | 0.209 | 0.028 |  | 0.009 |  |  |
|  |  | Riang ^c^ | India, Northeast | 48 | 0.148 | 0.503 | 0.253 | 0.023 | 0,000 | 0,000 | 0.012 | 0.061 |
|  |  | Tripperah ^c^ | India, Northeast | 51 | 0.143 | 0.459 | 0.272 | 0.035 | 0.016 |  |  | 0.075 |
| Austro-Asiatic |  | Cambodians ^a^ | Cambodia (collected in the USA) | 25 | 0.04 | 0.476 | 0.374 | 0.044 | 0,000 | 0.066 | 0,000 | 0,000 |
| Dravidian |  | Ambalakarer ^c^ | India, South | 50 | 0.26 | 0.537 | 0.087 | 0.013 | 0.039 | 0.043 | 0.02 | 0.001 |
| Austonesian |  | Ami ^a^ | Taiwan, East | 40 | 0.024 | 0.6 | 0.286 | 0.013 |  | 0.051 |  | 0.026 |
|  |  | Micronesians ^b^ | Micronesia | 37 | 0.137 | 0.517 | 0.226 | 0.033 | 0.029 | 0.034 | 0.024 |  |
| East Papuan |  | Melanesians (Nasioi) ^b^ | Solomon Islands, Bougainville Island | 24 | 0.142 | 0.604 | 0.207 |  | 0.025 | 0.022 |  |  |

^a^ Data from [7].

^b^ Data from the ALFRED database (http://alfred.med.yale.edu/alfred/index.asp) (Kidd, unpublished).

^c^ Data from [9].

^d^ For detailed language classification see http://www.ethnologue.com
